# Antimicrobial Effect of Platelet-Rich Plasma against *Porphyromonas gingivalis*

**DOI:** 10.1155/2019/7329103

**Published:** 2019-05-13

**Authors:** Thuy Anh Vu Pham, Thao Thi Phuong Tran, Ngan Thi My Luong

**Affiliations:** ^1^Department of Periodontology, Faculty of Odonto-Stomatology, University of Medicine and Pharmacy at Ho Chi Minh City, 652 Nguyen Trai Str., District 5, Ho Chi Minh City, Vietnam; ^2^Faculty of Odonto-Stomatology, Hong Bang International University, 120 Hoa Binh Str., District Tan Phu, Ho Chi Minh City, Vietnam; ^3^Department of Plant Biotechnology and Biotransformation, Faculty of Biology, University of Science, Vietnam National University Ho Chi Minh City, 227 Nguyen Van Cu Street, District 5, Ho Chi Minh City, Vietnam

## Abstract

**Aim:**

The aim of our study was to evaluate whether there was a difference in antimicrobial effect between the PRP of healthy volunteers and that of patients with chronic periodontitis against *P. gingivalis,* which was fresh cultured from subgingival plaque.

**Methods:**

Subgingival plaque of patients with moderate and severe chronic periodontitis was collected to isolate *P. gingivalis*. The PRP of four individuals with healthy periodontium and four patients with moderate and severe periodontitis were collected with a specific kit using a two-centrifuge procedure, and then, the antibacterial properties against *P. gingivalis* were tested, through their minimum inhibitory concentration (MIC), adhesion resistance assay, and biofilm susceptibility assay.

**Results:**

*P. gingivalis* was successfully isolated from the subgingival plaque of the 21st patient. The round, smooth, and black colony appeared in the agar disk after 7–10 days of incubation under anaerobic conditions. Bacterial identification was performed by MALDI-TOF and confirmed by PCR. All PRP samples tested showed the ability to inhibit *P. gingivalis* growth. The MIC value (expressed as fraction of PRP) was 1/2, and PRP prevented *P. gingivalis* attachment on the disk surface. However, PRP did not have a strong effect on the suppression of *P. gingivalis* biofilm.

**Conclusion:**

PRP of individuals with healthy periodontium and chronic periodontitis patients showed antibacterial properties against *P. gingivalis.* This material can become an adjunct to periodontal treatment.

## 1. Introduction

Periodontitis is a complex, multifactorial, and multibacterial disease characterised by the destruction of support tissues. While subgingival plaque contains more than 700 types of bacteria, studies have shown that *P. gingivalis*, an anaerobic bacterium, is one of the major pathogens of periodontitis [1]. Periodontal treatments include surgical and nonsurgical procedures with the goal of removing plaque and pathogenic bacteria. However, even when the infection control measures are tightened, the bacteria can still survive and invade the underlying tissue [2]. Therefore, using antimicrobial adjuncts is important for the success of healing and the prevention of chronic conditions.

Platelet-rich plasma (PRP) was recently developed as a method for healing improvement and the regeneration of tissues. This material is used in many medical fields such as prosthetics, dermatology, aesthetics, dentistry, and facial surgery [3]. Platelet alpha-granules have the ability to release large numbers of peptides and growth factors. This helps the PRP to influence and promote the biological processes required for healing and tissue regeneration [3]. In addition, the antimicrobial effect of this material has been reported in several studies on oral bacteria with mixed results [4, 5]. For periodontal pathogens, especially *P. gingivalis*, just a few studies have examined the effects of PRP on the growth and development of these bacteria. In addition, in most of the available studies, PRP was collected from the blood of healthy volunteers, and commercial bacterial strains were tested [3, 6, 7]. In this study, we collected the PRP of healthy individuals and patients with moderate and severe periodontitis and then isolated fresh *P. gingivalis* from the subgingival plaque of patients to evaluate whether PRP still exhibited antimicrobial properties.

## 2. Materials and Methods

### 2.1. Subjects

For the isolation of *P. gingivalis*: subgingival plaque was obtained from a patient with moderate to severe chronic periodontitis according to the classification criteria of the American Association of Periodontology 2015 [8] who visited the Faculty of Odonto-Stomatology, University of Medicine and Pharmacy at Ho Chi Minh City, Vietnam, from 9/2017 to 12/2017.

For PRP procedures: 4 patients (2 males and 2 females, mean age: 55.25 ± 8.50) with moderate to severe chronic periodontitis and 4 volunteers (2 males and 2 females, mean age: 22.75 ± 2.75) with healthy periodontium whose probing depth was less than or equal to 3 mm and who showed no clinical loss attachment.

All subjects were systemically healthy, nonsmokers, with no symptoms of infection and took no antibiotics for at least 3 months before the experiments. All subjects were informed about the objective of the study and voluntarily participated. Ethical approval was obtained from the Ethical Review Committee of the University of Medicine and Pharmacy at Ho Chi Minh City.

### 2.2. Procedures

#### 2.2.1. Isolation of *P. gingivalis*

Specimens were collected until the appearance of *P. gingivalis*. The sampling procedure was performed according to the study of Doan et al. [9]. Briefly, after removing supragingival plaque, sterile paper points were inserted to the depth of each periodontal pocket sampled and retained for 10°s. These paper points were then transferred to 2 ml Eppendorf tubes containing Wilkins–Chalgren Anaerobic Broth Base (Himedia-M863). Samples were processed immediately. After dispersing bacteria with a vortex mixer for 45 seconds and ten-fold serial dilution, 0.1 ml of aliquots from 10^0^ to 10^3^ were inoculated onto blood agar plates containing 0.005 g/L hemin and 0.0005 g/L menadione. After incubation at 37°C for 7–10 days under anaerobic conditions, the identification of *P. gingivalis* isolates was carried out by the MALDI-TOF method. After determining the strains of *P. gingivalis*, a colony was held in a TE (Tris-EDTA) solution and sent for sequencing by PCR with specific primers, as suggested by Tran and Rudney [10]. Finally, bacterial strain was stored by freezing in a glycerol broth.

#### 2.2.2. Platelet-Rich Plasma Preparation

Here, 25.5 ml venous blood was used to prepare PRP according to the guidelines of the New-PRP Pro Kit (Geneworld Ltd., HCM, Vietnam). Briefly, whole blood was centrifuged at 2000 rpm for 10 minutes to obtain plasma. A second centrifugation step was then performed at 3500 rpm in 5 minutes, and the platelet poor plasma (PPP) layer was discarded. The final PRP concentrate was activated (by 100 *μ*l of 20% CaCl_2_), immediately diluted with cultured broth for antimicrobial assays.

#### 2.2.3. Minimum Inhibitory Concentration Determination

We used the broth microdilution method to determine the MIC [11], through double serial dilutions. Chlorhexidine gluconate at 0.12% was used as positive control and culture medium without adding PRP as a negative control. After incubation under anaerobic conditions for 24 hours at 37°C, MIC was determined using 0.015% resazurin.

#### 2.2.4. Bacterial Adhesion Assay

The bacterial adhesion and biofilm susceptibility assays were performed according to the protocol of Yang et al. [7]. Briefly, in our study, a total volume of 180 *μ*l of 50% (v/v) PRP in cultural medium was added to each well of the microtiter plate. Bacterial broth without PRP addition was used as a control. After anaerobic incubation for 24 hours at 37°C, bacterial attachment in the well bottom was stained by 0.1% crystal violet, and we measured the absorbance at 595 nm.

#### 2.2.5. Biofilm Susceptibility Assay

*P. gingivalis* in culture medium was added to each well of the microtiter plate and incubated in an anaerobic chamber at 37°C for 48 hours. After that, each well was replaced with 50% (v/v) PRP in cultural medium. Cultural medium alone served as a control. After incubating for 2 hours, staining with crystal violet was performed, and samples were read at 595 nm.

### 2.3. Statistical Analysis

The data are presented as the mean ± SD and were analysed using software Stata 13.0 (StataCorp, LLC, USA). Statistical differences were evaluated by the Wilcoxon rank sum test. A *P* < 0.05 was considered statistically significant.

## 3. Results

### 3.1. Bacterial Isolation

*P. gingivalis* was successfully isolated from the subgingival plaque of the 21st patient. It grew anaerobically on media containing lysed blood with dark pigmentation. These 1-2 mm colonies were rounded, smooth, and convex. After identification by MALDI-TOF, comparison of PCR sequencing results with the GenBank database (https://www.ncbi.nlm.nih.gov/BLAST/) confirmed the result. We have used 16S rRNA gene sequencing to identify bacterial strain in this study.

### 3.2. PRP Preparation

The study obtained 4 mL to 5 mL of PRP from each participant. Platelet concentrations in PRP after enrichment increased by approximately 2- to 5-fold compared to whole blood (Table 1). No statistical difference was found between platelet counts of the healthy group and the periodontitis group.

### 3.3. MIC

The columns with no colour change scored as the MIC value. PRP from all participants, in both the healthy and periodontitis groups, showed the ability to inhibit bacteria at a minimum concentration of 1/2, with the PRP from one healthy subject being able to inhibit *P. gingivalis* at a value of 1/4 (Table 2).

### 3.4. Bacterial Adhesion Assay

**Figure 1:**
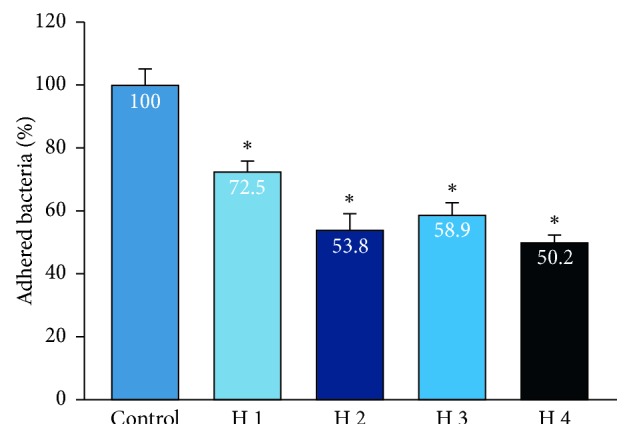
Bacterial adhesion resistance of PRP from healthy volunteers. Data show the percentage adhesion of the control group, *n* = 4. ^*∗*^indicates the Wilcoxon nonparametric analysis showed a significant bacterial adhesion reduction between the PRP of each subject and the control group (*P* < 0.05).

**Figure 2:**
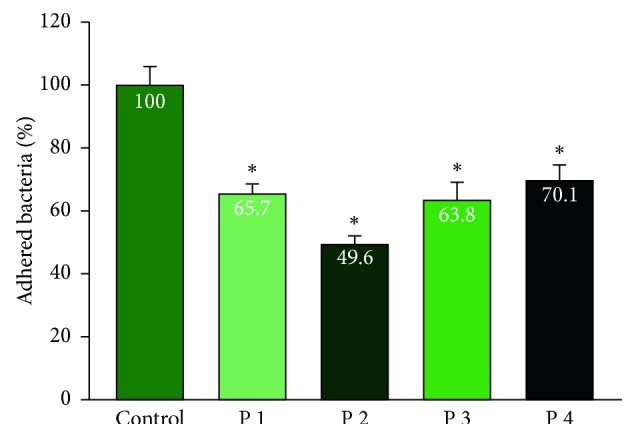
Bacterial adhesion resistance of PRP of patients with periodontitis. Data show the percentage adhesion of the control group, *n* = 4. ^*∗*^indicates the Wilcoxon nonparametric analysis showed a significant bacterial adhesion reduction between PRP of each subject and the control group (*P* < 0.05).

The results showed that all PRP samples reduced bacterial adhesion to the bottom of the plate when compared to the control group with *P* < 0.05. There was no significant difference in bacterial adhesion reduction between the PRP of the healthy and periodontitis groups (Figures 1 and 2).

### 3.5. Biofilm Susceptibility Assay

**Figure 3:**
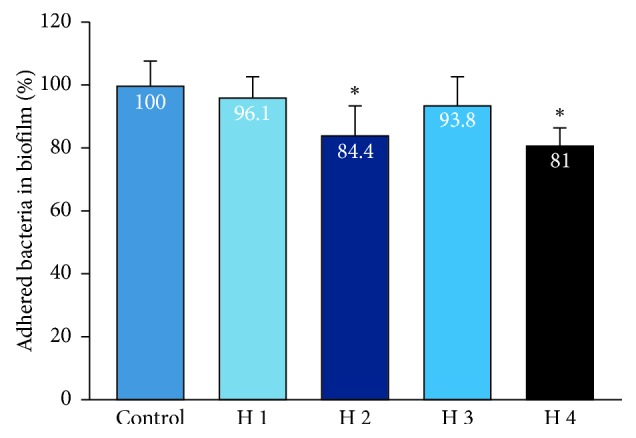
Bacterial biofilm resistance of PRP of the healthy group. Data show the percentage of bacteria in the biofilm of the control group, *n* = 4; the error bar represents the standard deviation. ^*∗*^indicates the Wilcoxon nonparametric test showed significant differences in mean biofilm reduction between the PRP of subjects and the control group (*P* < 0.05).

**Figure 4:**
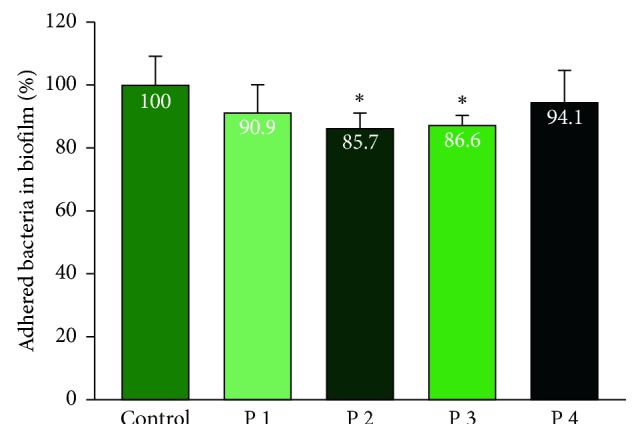
Bacterial biofilm resistance of PRP of the periodontitis group. Data show the percentage of bacteria in the biofilm of the control group, *n* = 4; the error bar represents the standard deviation. ^*∗*^indicates the Wilcoxon nonparametric test showed significant differences in mean biofilm reduction between the PRP of subjects and the control group (*P* < 0.05).

We evaluated the ability of pure PRP against adhered *P. gingivalis*. Some PRP samples were able to reduce the amount of adhered *P. gingivalis* compared to the control group, but others could not. There was no significant difference in biofilm resistance between the PRP of two groups (Figures 3 and 4).

## 4. Discussion

Periodontal disease is a chronic inflammatory disease that causes tooth loss, and *P. gingivalis* is thought to be its major pathogen [1]. Although the detection and quantification of *P. gingivalis* has been gradually replaced by modern methods such as PCR, bacterial culture still plays an essential role in providing the source of bacteria for conducting comparative studies [12, 13]. While available commercial bacterial strains shortened the culture time, varying from 2 to 4 days, fresh isolates of *P. gingivalis* from subgingival samples required longer culture times [14, 15]. Our study took 7–10 days to observe the presence of *P. gingivalis* on blood agar plates. This was consistent with many previous studies [14, 15]. In our study, up to the 21st patient sample, *P gingivalis* colonies did appear. In spite of the increased rate of detection of *P. gingivalis* through molecular and immunohistochemistry methods, this does not correspond to the success rate of culture of this microorganism because of a number of factors [16, 17]. Concerning identification, according to Clark et al., the MALDI-TOF method provides a powerful and accurate tool to quickly identify bacteria; through the use of MALDI-TOF, *Porphyromonas* isolates can be distinguished [18].

PRP in our study was collected from two healthy populations and periodontitis patients using a two-centrifuge procedure. This is the classic method applied in studies using PRP [19]. In Table 1, the platelet counts in the two groups after enrichment increased from 2.7 to 5.1 times that in the total blood, which is consistent with many studies [4, 11, 20]. The preenrichment platelets (total blood) between the two groups were not significantly different. The same thing happened with the platelet counts of the two groups obtained after centrifugation. This was consistent with the studies of Wang et al. [21] or Kumar et al. [22]. However, many previous studies have shown that patients with periodontal inflammation have significant changes in blood parameters when compared to healthy ones [23, 24]. *P. gingivalis* is not only found in the blood but also in atherosclerotic plaques, and a study by Lourbakos et al. showed that gingipains of this bacterium play a role in platelet aggregation [25]. Thus, platelets not only have an effect on the bacteria but also have the virulence factors of the bacteria that also stimulate and affect the response of platelets [26].

MIC is defined as the minimum inhibitory concentration at which an active agent can inhibit the visible development of microorganisms and is thought to be the gold standard for determining bacterial susceptibility. The results in Table 1 showed that the value of MIC of all PRP samples was 1/2, except for one healthy volunteer, who had an MIC of 1/4. The latter result corresponded to the results of Yang et al., in which the MIC values of PRP against *P. gingivalis, A. actinomycetemcomitans,* and *F. nucleatum* were 1/4, 1/8, and 1/2, respectively. However, with the remaining subjects in our study, *P. gingivalis* was only inhibited by a value of MIC 1/2 [7]. This difference is probably due to the nature of freshly cultured *P. gingivalis*, which may have greater virulence than the PRP collected from different subjects. Studies by Drago et al. showed that the MIC levels of PRPs ranged from 1/8 to 1/16 when tested on *E. faecalis, S. oralis, S. agalactiaea,* and *S. aureus* [11]. Meanwhile, Rózalski et al. found that the combination of PRP with oxacillin reduced the use of antibiotics from 0.25 mg/L to 0.19 mg/L. Similarly, the amount of vancomycin reduced from 1.0 mg/L (control group) to 0.5 mg/L (with PRP). This result confirmed the synergistic effects of the antimicrobial proteins of platelets with antibiotics such as nafcillin, ampicillin, and vancomycin [27]. The capacity of *P. gingivalis* inhibition of PRP in our results was similar to those of other studies using the agar diffusion [6, 20]. In addition, Aggour and Gamil conclude that there is no difference in bacterial resistance between the healthy group and periodontitis group [20].

Adhesion is the first step of the infection process. Frimbia of *P. gingivalis* help bacteria to attach to the surface of teeth, periodontal tissue, epithelial cells, fibroblasts, red cells, and white blood cells and make them bond together [1]. According to Figures 1 and 2, PRP showed a significant reduction in the number of bacteria compared with the control group without PRP supplementation. The study by Yang et al. also showed similar results, with a significant reduction in the adhesion of PRP material [7]. However, the PRP concentration in this author's study was 12.5% while our concentration was 50%. With the use of fresh isolates of *P. gingivalis*, as well as high minimal inhibitory concentrations, increased concentrations clearly showed bacterial reduction with small amounts of PRP available. In addition, in both patients with periodontal diseases and healthy individuals, PRP demonstrated a reduced ability against adhered *P. gingivalis*. This ability may come from peptides present in platelets that inhibit bacterial adhesion with a partially coagulant and inert action. In fact, *P. gingivalis* combines with other bacteria to attach to the periodontal tissues and tooth surface. Bazaka's research [28] showed that *Streptococci* bacteria first bound to the surface of the teeth and created secondary zones for pathogenic bacteria such as *F. nucleatum* or *P. gingivalis*. Thus, when investigated alone, the adhesion ability of *P. gingivalis* may be different than in the oral environment. The difference in percent reduction of bacteria in each patient can be explained by the platelet diversity properties. The PRP of each patient has different platelet levels, which may vary in size and composition, in particular the alpha granule composition, which contains growth factors and antibacterial peptides. Differences in age also alter platelet aggregation, thereby affecting the homogeneity of the effect of platelet-rich plasma.

Biofilm is defined as an organised microbial community that is encapsulated in the extracellular matrix and attached to living or nonbiological surfaces. This is an adaptive strategy for the survival of bacteria in host environments, including humans [29]. Figures 3 and 4 show that the biofilm destruction effect of PRP varied among each subject, and, compared to the adhesion test, and a lower rate of detached bacterial number observed as the biofilm was firmly attached to the bottom of the plate. PRP in some subjects in our study showed biofilm resistance, which is similar to the study by Yang et al. when PRP reduced an average of 25% of the *P. gingivalis* bacteria in the biofilm compared with the control group. However, the author used only one PRP sample to perform the experiment [7]. In addition, one of the most important characteristics of biofilms is their ability to withstand the increase in antimicrobial agents, and according to Macià et al., the biofilm can tolerate antibiotics and antibacterial agents more than 100–1,000 times higher than planktonic bacteria. There are many methods for testing biofilm resistance [29]. We used a 96-well flat plate method to test for *P. gingivalis*. The advantage of this method is its rapidity and repeatability, but one drawback with using crystalline violet is that it does not distinguish living and dead cells from the biofilm. Testing on biofilm was important for clinical practice, especially in periodontitis treatment. Experimental results showed the importance of mechanical measures in the treatment of periodontitis. Scaling and root debridement, according to Tariq et al., are the “gold standard” of nonsurgical periodontal treatment and are indispensable for obtaining a smooth surface that is resistant to bacterial adhesion. When bacteria cannot adhere, their ability to create biofilm and multiply their toxicity decreases, contributing to a prolonged therapeutic effect [30]. At the same time, the combination of mechanical methods and antimicrobial chemical agents is used to increase the effectiveness of the treatment of periodontitis.

In our study, PRP showed antimicrobial properties against fresh isolates of *P. gingivalis* through minimal inhibitory concentration and adhesion resistance tests. Small sample sizes do not allow us to confirm the difference in the antimicrobial effects of PRP between healthy subjects and patients with periodontitis; however, the PRP of patients with periodontitis also exhibited the ability to reduce the levels of *P. gingivalis*, confirming the potential use of the patient's own blood in the treatment of postoperative infection.

## 5. Conclusion

Through the results in this study, PRP also showed bacteriostatic effects against a major periodontal pathogen, *P. gingivalis*. It is possible to use this material as an adjunct with antibiotics to reduce the amount of bacteria, as well as the best mechanical treatment to prevent bacterial adhesion and biofilm formation on periodontal tissue.

## Figures and Tables

**Table 1:** Platelet count in whole blood and in PRP of participants (×10^6^ platelets/mL).

Platelets count	Whole blood	PRP
H 1	266.2 ± 4.1	1102.4 ± 6.5
H 2	302.6 ± 3.3	1057.4 ± 41.6
H 3	296.4 ± 7.5	1000.6 ± 12.4
H 4	217.2 ± 1.7	574.6 ± 20.5
P 1	249.6 ± 3.4	773.6 ± 3.1
P 2	235 ± 3.7	1187 ± 4.2
P 3	280.2 ± 2.8	896.6 ± 4.1
P 4	179.4 ± 1.7	836.4 ± 5.8
	*P* value > 0.05^*∗*^	*P* value > 0.05^*∗∗*^

^*∗*^  *and* ^*∗∗*^ indicates no statistical significant difference between two groups (in same column) using the Wilcoxon nonparametric test, H: healthy volunteers, and P: patients with chronic periodontitis.

**Table 2:** Minimum inhibitory concentration (MIC) of PRP against *P. gingivalis* (represented as fraction of PRP and platelets concentration).

PRP	Healthy volunteer 1	Healthy volunteer 2	Healthy volunteer 3	Healthy volunteer 4	Patient 1	Patient 2	Patient 3	Patient 4
MIC	1/4 (275.6 × 10^6^/ml)	1/2 (528.7 × 10^6^/ml)	1/2 (500.3 × 10^6^/ml)	1/2 (287.3 × 10^6^/ml)	1/2 (386.8 × 10^6^/ml)	1/2 (593.5 × 10^6^/ml)	1/2 (448.3 × 10^6^/ml)	1/2 (418.2 × 10^6^/ml)

## Data Availability

The data used to support the findings of this study are available from the corresponding author upon request.
